# Diffuse Large B-Cell Lymphoma of the Left Upper Extremity Mimicking a Sarcoma

**DOI:** 10.7759/cureus.15588

**Published:** 2021-06-11

**Authors:** Raheel S Siddiqui, Debra Ferman, Sandeep Tuli, M. Margaret Kemeny

**Affiliations:** 1 Department of Internal Medicine, Icahn School of Medicine at Mount Sinai, New York City Health and Hospitals/Queens, Jamaica, USA; 2 Department of Oncology, Queens Cancer Center, Icahn School of Medicine at Mount Sinai, New York City Health and Hospitals/Queens, Jamaica, USA; 3 Department of Radiology, Icahn School of Medicine at Mount Sinai, New York City Health and Hospitals/Queens, Jamaica, USA; 4 Department of Surgical Oncology, Queens Cancer Center, Icahn School of Medicine at Mount Sinai, New York City Health and Hospitals/Queens, Jamaica, USA

**Keywords:** diffuse large b cell lymphoma, sarcoma, upper extremity lymphoma, soft tissue lymphoma, small blue cell neoplasm

## Abstract

Diffuse large B-cell lymphoma (DLBCL) can arise in both lymph nodes and extranodal sites. DLBCLs rarely present in the soft tissue of the upper extremity. We report a case of a 64-year-old woman who presented with a large left upper arm mass and underwent surgical resection under the presumptive diagnosis of sarcoma but the final pathology showed DLBCL. Sarcomas are common malignant tumors of the soft tissue of the extremities, but lymphomas also occasionally present as a soft tissue mass. It is important to keep lymphomas in mind in order to avoid unnecessary surgical excisions.

## Introduction

Diffuse large B-cell lymphoma (DLBCL) is the most common subtype of non-Hodgkin lymphoma (NHL) and accounts for 31% of all cases of NHL in the world [1]. The median age for DLBCL is between the sixth and seventh decade of life. Diffuse Large B-cell Lymphomas can arise in an extranodal organ in up to 30% of cases with the most common sites being gastrointestinal tract, skin and soft tissue, bone or genitourinary tract [2]. DLBCLs are rare in the soft tissue of the upper extremity. Soft tissue sarcomas are malignant tumors of mesenchymal origin that may occur anywhere in the body, with extremities accounting for 60% of cases [3]. We report a case of DLBCL of the soft tissue of the left upper extremity that was initially thought to be a sarcoma based on clinical features, imaging and core biopsy, but was later diagnosed to be DLBCL after surgical excision of the mass.

## Case presentation

**Figure 1:**
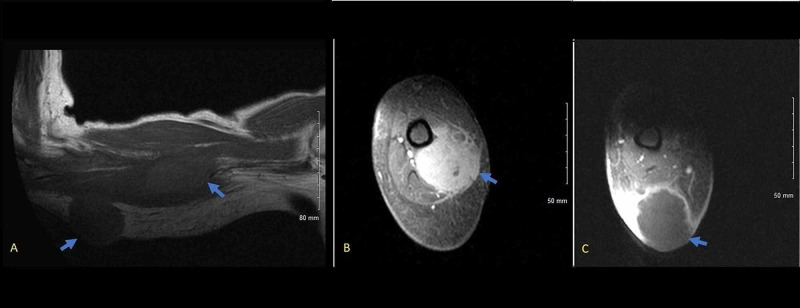
Images of the MRI of left upper extremities. (A) shows both proximal and distal mass in coronal section, (B) shows proximal deep soft tissue mass between biceps and triceps in left arm, (C) shows distal soft tissue subcutaneous mass.

**Figure 2:**
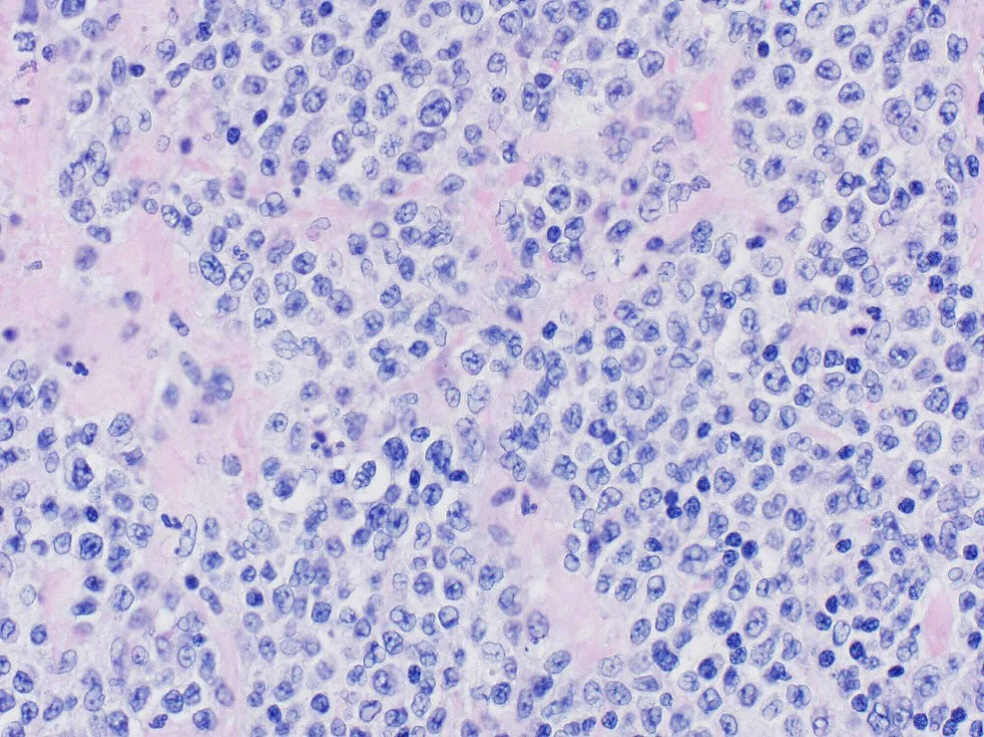
Small blue cell neoplasm on excisional biopsy that led to the diagnosis of DLBCL after immunohistochemically staining. DLBCL: diffuse large B-cell lymphoma.

**Figure 3:**
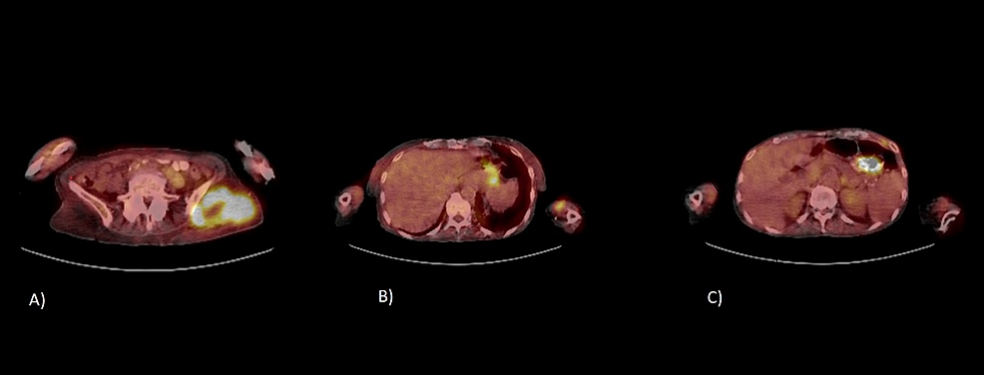
(A) shows avid lesion in left gluteal region, (B) shows avid lesion in stomach, (C) shows avid lesion in small intestine.

A 64-year-old woman presented to the emergency room with a five-month history of an enlarging mass in the left arm and associated pain and redness for two weeks. The patient had a history of scleroderma for 15 years with five years of interstitial lung disease, calcified nodules and anemia of chronic disease. She experienced six months of increased fatigue, anorexia and unintentional weight loss of 20 pounds without any fevers or night sweats. Physical examination showed a tender, non-fluctuant, 5 cm x 5 cm immobile subcutaneous mass with overlying skin excoriations and minimal surrounding erythema. Multiple old raised calcified skin nodules on the wrist, forearm, elbow and thigh were noted. No cervical, axillary or inguinal lymphadenopathy noted. MRI of the left upper extremity with contrast showed a 4.7 cm x 3.4 cm x 3.6 cm mass in the middle-upper arm deep soft tissue between the biceps and triceps muscle and a second 4.9 cm x 4 cm x 4.5 cm mass in the subcutaneous tissue of the distal posterior part of the arm (Figure 1). Core needle biopsy of the mass showed a fragment of completely necrotic tissue with necrotic cells suggestive of small blue cell neoplasm. The patient underwent surgical excision of the left arm masses with the presumptive diagnosis of sarcoma. Surgical findings included a distal superficial necrotic mass which was easily excised and a proximal deeper mass which involved muscle, surrounding nerves and vessels, and was adherent to the bone. This deeper mass was completely excised in pieces. Frozen section of the biopsy showed a small blue cell neoplasm with extensive necrosis in both masses (Figure 2). Immunohistochemical (IHC) studies established the diagnosis of Diffuse Large B-cell Lymphoma. IHC was positive for CD45, CD 20, BCL2, and partial BCL6. Fluorescence in situ hybridization analysis of the neoplastic cells were positive for BCL-6 rearrangement in 58% of cells and negative for a rearrangement involving MYC and BCL2. Postoperative positron emission tomography (PET) scan showed an intense avid soft tissue mass in the left gluteal region (standard uptake value [SUV] max 11.8), increased uptake in stomach and thick-walled loops of small bowel (SUV max 10.1) (Figure 3). Borderline to enlarged mediastinal and hilar lymph nodes with SUV max ranging 8.5-9.0 along with scattered patchy lung opacities with varied uptake were reported. The patient was diagnosed with Stage IV DLBCL and treated with R-CHOP (Rituximab- cyclophosphamide, vincristine, and prednisone) therapy.

## Discussion

DLBCL is extremely uncommon in the soft tissue of the upper extremity and a few cases have been reported in the literature, where they were initially mistaken to be sarcomas and ultimately diagnosed as DLBCL only after biopsy. Mamorska-Dyga et al. reported a case of right arm violaceous fleshy tumor above the antecubital fossa that was initially thought to be a sarcoma based on physical examination and imaging results but after biopsy was diagnosed to be DLBCL [4]. Mayo et al. reported a case of DLBCL of the subcutaneous tissue of the right shoulder with completely normal skin and absent lymph nodes that underwent complete surgical excision under the impression of sarcoma [5]. In addition to DLBCLs, other types of lymphoma may also involve the soft tissue. Knowles and Serpell (2003) carried out a review of cases of lymphomas that presented as a soft tissue mass in the absence of nodal involvement that looked like soft-tissue sarcoma. The pathology for these soft tissue lymphomas were nodular lymphocyte predominant, mixed cellularity and nodular sclerosing Hodgkin lymphoma, small lymphocytic lymphoma, DLBCL, follicular small cell lymphoma, follicular large cell lymphoma and follicular mixed cell lymphoma [6]. Gupta et al. reported a case of left inguinal swelling and ulcer that mimicked sarcoma based on history and clinical presentation but biopsy established the diagnosis of primary cutaneous B-cell lymphoma [7]. Most patients were diagnosed after biopsy, but in our patient the first core biopsy was not diagnostic and showed necrotic cells suspicious for small blue cell neoplasm.

Small blue cell neoplasms encompass a broad spectrum of neoplasms such as lymphoma, mesenchymal chondrosarcoma, rhabdomyosarcoma, small cell osteosarcoma, Ewing sarcoma/primitive neuroectodermal tumor, melanoma, squamous cell carcinoma, olfactory neuroblastoma, pituitary adenoma or plasmacytoma and often pose diagnostic challenges to surgical pathologists. These tumors often share histological and immunophenotypical features and the clinician must depend on tumor site of origin, clinical findings and imaging studies for accurate diagnosis [8]. Sarcomas are typically treated by surgery and radiotherapy whereas the primary mode of treatment for lymphomas is chemotherapy [3,9]. Imaging such as CT scan or PET scan should be done to assess for lymphadenopathy and other extranodal organ system involvement suggestive of lymphoma. According to one series, core needle biopsy not only required more immunohistochemical tests but was unable to yield a definitive diagnosis in 8.3% of patients as compared to 2.8% who underwent surgical excisional biopsy (p-value: 0.0003) [10]. This case underscores the importance of keeping lymphoma in the differential diagnosis of sarcoma of the soft tissue of the extremity when initial core biopsy shows small blue cell neoplasm in order to avoid unnecessary surgical excision and delay in diagnosis and appropriate treatment.

## Conclusions

We present the case of a small blue neoplasm of the upper extremity that clinically appeared to be sarcoma but then was found to be diffuse large B cell lymphoma. Small blue round neoplasm on core biopsy may be seen in many solid tumors and hematological malignancies, and warrants a thorough immunohistochemical and clinical workup to establish the correct diagnosis. On core biopsy, lymphoma may be missed if there is a low index of suspicion. Lymphoma should always be considered in extremity tumors thought to be sarcoma.

## References

[1] Martelli M, Ferreri AJ, Agostinelli C, Di Rocco A, Pfreundschuh M, Pileri SA (2013). Diffuse large B-cell lymphoma. Crit Rev Oncol Hematol.

[2] Ting CY, Gan GG, Bee-Lan Ong D, Tan SY, Bee PC (2020). Extranodal site of diffuse large B-cell lymphoma and the risk of R-CHOP chemotherapy resistance and early relapse. Int J Clin Pract.

[3] Hoefkens F, Dehandschutter C, Somville J, Meijnders P, Van Gestel D (2016). Soft tissue sarcoma of the extremities: pending questions on surgery and radiotherapy. Radiat Oncol.

[4] Mamorska-Dyga A, Ronny FM, Puccio C, Islam H, Liu D (2016). A rare case of the upper extremity diffuse large B-cell lymphoma mimicking soft tissue sarcoma in an elderly patient. Stem Cell Investig.

[5] Mayo J, Bogenberger K, Raj T, Reha J (2019). Subcutaneous mass concerning for sarcoma: a peculiar presentation of diffuse large B-cell lymphoma. BMJ Case Rep.

[6] Knowles B, Serpell JW (2003). Extra-nodal lymphoma presenting as a mimic of soft-tissue sarcoma. ANZ J Surg.

[7] Gupta P, Agarwal P, Ahuja A, Durga CK (2018). Primary cutaneous non-Hodgkin's lymphoma, clinically mimicking a soft tissue sarcoma. Cytojournal.

[8] Thompson LD (2017). Small round blue cell tumors of the sinonasal tract: a differential diagnosis approach. Mod Pathol.

[9] Chiappella A, Castellino A, Nicolosi M, Santambrogio E, Vitolo U (2017). Diffuse large B-cell lymphoma in the elderly: standard treatment and new perspectives. Expert Rev Hematol.

[10] Johl A, Lengfelder E, Hiddemann W, Klapper W (2016). Core needle biopsies and surgical excision biopsies in the diagnosis of lymphoma-experience at the Lymph Node Registry Kiel. Ann Hematol.

